# Bioresource-Functionalized Quantum Dots for Energy Generation and Storage: Recent Advances and Feature Perspective

**DOI:** 10.3390/nano12213905

**Published:** 2022-11-05

**Authors:** Seyyed Mojtaba Mousavi, Seyyed Alireza Hashemi, Masoomeh Yari Kalashgrani, Darwin Kurniawan, Ahmad Gholami, Wei-Hung Chiang

**Affiliations:** 1 Department of Chemical Engineering, National Taiwan University of Science and Technology, Taipei City 106335, Taiwan; 2 Nanomaterials and Polymer Nanocomposites Laboratory, School of Engineering, University of British Columbia, Kelowna, BC V1V 1V7, Canada; 3 Biotechnology Research Center, Shiraz University of Medical Science, Shiraz 71468-64685, Iran

**Keywords:** bioresource, quantum dots, energy generation, energy storage

## Abstract

The exponential increase in global energy demand in daily life prompts us to search for a bioresource for energy production and storage. Therefore, in developing countries with large populations, there is a need for alternative energy resources to compensate for the energy deficit in an environmentally friendly way and to be independent in their energy demands. The objective of this review article is to compile and evaluate the progress in the development of quantum dots (QDs) for energy generation and storage. Therefore, this article discusses the energy scenario by presenting the basic concepts and advances of various solar cells, providing an overview of energy storage systems (supercapacitors and batteries), and highlighting the research progress to date and future opportunities. This exploratory study will examine the systematic and sequential advances in all three generations of solar cells, namely perovskite solar cells, dye-sensitized solar cells, Si cells, and thin-film solar cells. The discussion will focus on the development of novel QDs that are economical, efficient, and stable. In addition, the current status of high-performance devices for each technology will be discussed in detail. Finally, the prospects, opportunities for improvement, and future trends in the development of cost-effective and efficient QDs for solar cells and storage from biological resources will be highlighted.

## 1. Introduction

In the coming years, as fossil fuel consumption increases, so will energy demand. This problem puts a lot of pressure on renewable energy sources. At the same time, the reduction in renewable energy sources leads to environmental pollution, reduction in natural resources, and global warming [1,2,3]. Therefore, it is necessary to develop new methods of energy generation and storage for the times when they are needed. Lithium batteries and supercapacitors will be among the energy storage devices of the future [4,5,6,7]. In recent decades, a new type of capacitor, supercapacitors/microcapacitors (SCs/MSCs), has been developed. Supercapacitors are among the energy storage devices used in a variety of applications, such as hybrid vehicles, personal electronic devices, digital telecommunication systems, energy storage in conjunction with other energy systems, such as solar cells, backup power in computers, and in the electrical industry [8,9,10]. One way to optimize the properties of energy storage systems is to develop new materials for use as electrode materials in supercapacitors, batteries, and solar cells [11,12,13,14]. Quantum materials, such as carbon quantum dots (CQDs) and graphene quantum dots (GQDs), play an important role in supercapacitors, batteries, and solar cells due to their good electrical conductivity, low cost and availability, biocompatibility, and good chemical stability, and one of the latest methods is to obtain QDs from bioresources. Another important feature of CQDs/GQDs for solar cell applications is their excellent photoreaction [15,16,17,18,19]. Due to the synthesis of GQDs by micro-organisms, such as bacteria [20], fungi, and yeasts [21], they are a very suitable option for the development of the next generations of electronic and optical components, fuel cells, solar cells, lithium batteries, and bioresources [22,23,24]. It is worth noting that research on quantum dots from biological sources for energy generation and storage is limited. Therefore, we tried to study a large part of the applications of quantum dots in energy generation and storage, the applications of biological resources in energy generation and storage separately, and a combination of quantum dots derived from biological resources in energy generation and storage. This review article will enable researchers to use quantum dots from biological sources for energy-related applications in the near future. Therefore, this review provides an overview of recent progress and prospects for the characterization of bioresource-derived QDs for energy generation and storage. The synthesis of QDs using bioresources, e.g., micro-organisms, such as fungi, bacteria, and yeasts, is being extensively studied. This review shows the versatile applications of QDs derived from biological sources for energy generation and storage, which are very promising, such as supercapacitors/microcapacitors, batteries, and solar cells (perovskite solar cells and organic and inorganic hybrid solar cells).

## 2. Bioresource-Derived QDs

Bioresources include both plant and animal materials, and each of these materials can be converted into renewable energy through the use of bioresources. In general, bioresources can be divided into three types: micromolecules derived from bioresources (e.g., citric acid and glucose), components of bioresources (e.g., cellulose, hemicellulose, lignin, etc.), and natural bioresources (e.g., straw and crab shells) [25]. Various types of bioresources can be used as carbon sources in QDs, e.g., waste streams. In addition, products that do not fall into the category of waste streams can also be used as carbon sources. Waste bioresources, such as leaves or food waste, are considered the most environmentally friendly, facilitate waste management, and are often less expensive. Although these bioresources cannot be considered waste, they are still environmentally friendly because they do not contribute to waste management. However, they are less environmentally friendly than those whose waste they can dispose of. Compared to waste bioresources, this type of bioresource is often more expensive, but can be better purified, so its synthesis can be simplified. In this section, we will describe the advantages of using bioresources to produce quantum dots with minimal environmental impact. In terms of their photoluminescence (PL) emission, optical properties, excellent conductivity, and broad spectral absorption, these dots share some similarities with chemical QDs, especially in terms of their chemical stability and broad optical absorption spectrum. Their properties include nonblinking, resistance to photobleaching, broad excitation wavelengths, and the ability to tune synthesis parameters, including size, shape, composition, internal structure, and surface chemistry, to emit at a specific wavelength [26,27]. These materials are not only more environmentally friendly, but they are also potentially less toxic and biocompatible.

## 3. Synthesis of Bioresource-Derived QDs

Synthesis for obtaining QDs from bioresources with good electronic and optical properties is possible by a variety of precursors and methods. The properties, advantages, and disadvantages of each method are listed in Table 1. In addition to these methods, microwave hydrothermal, oxidation, solvent thermal, and reflux methods are less common synthesis methods. Chemical oxidation is one of the least colorful methods because strong acids are used to quantify the quantum resource, while the bioresource extraction method without heat or chemicals is one of the most environmentally friendly methods. The lack of control over the properties of the extracted points is one of the major drawbacks of the extraction method. From the analysis, microwave and hydrothermal methods are widely used for QD synthesis [28,29,30,31].

### 3.1. Hydrothermal Method

The hydrothermal method is one of the most popular methods for producing QDs quickly, cheaply, conveniently, and in an environmentally friendly manner, producing quantum dots at a high rate under aqueous conditions of high temperature and pressure. There are a number of reasons why this method is so popular, including ease of use, control, nontoxicity, and the fact that the polymer does not need to be passivated before use. Nevertheless, this method does not allow for precise control of size, and impurities may already be present at the time of the process [37,38,39,40,41,42].

A 2016 paper by Wang et al. reported that the authors developed a new environmentally friendly method for hydrothermal carbonization of papaya to synthesize CDs.

Papaya was heated at 200 °C for 5 h, filtered, and dialyzed for 72 h, after which an extraction procedure was carried out at 2–6 nm with a high quantum yield of 18.3%. The result was a blue CD PL with a size of 2–6 nm and a quantum yield of 18.3%. The researchers performed a series of experiments to show that the dots were suitable for imaging HeLa cells as well as for measuring the fluorescence of *E. coli* bacterial strains. However, it should be emphasized that no percentage yield was reported in the other research cited; therefore, it is impossible to draw a conclusion as to whether or not this reaction is effective with further magnification [38].

### 3.2. Microwave Synthesis

In microwave synthesis, it is possible to quantify organic substances using microwaves to produce QDs. The yield and quality of quantum dots increase with a short heating time, as this increases the quantum yield. It has the added advantage of being very efficient, saving time, and distributing heat evenly. However, it is also characterized by poor size control and microwave radiation, which can be harmful to human health on a large scale [32,33,34,35,36].

Bajpai et al. used milk powder containing casein as a carbon source in their study. A microwave oven can be used to heat a casein solution until it becomes semisolid, so that a semisolid solution is formed by the heating process. The residue from this process is then diluted with distilled water and centrifuged to remove all residues. The supernatant is collected and analyzed. The environmental friendliness and the fact that no harsh chemicals are used in this process make it an ideal alternative for those who want to protect the environment. Only a few steps are required to complete the process. Since this method does not use waste biomass to make the synthesis, but instead buys the biomass to make it in a lab, it is less environmentally friendly than the others. To determine if the casein points described in the paper are more economical than those created from dairy waste, both the casein points described in the paper and those created from dairy waste must be compared. To determine whether casein points are more economically advantageous, the casein points described in the paper must be compared. Note that these points have a high yield of 18.7%, which means they fall in the blue range [32].

## 4. Limiting Factors of Using Bioresources for the Synthesis of Quantum Dots

There are a limited number of applications for QDs compared to SCQDs due to their low quantum yield [50]. Quantum yields between 40% and 90% [51] can be achieved with SCQDs, while QDs usually achieve quantum yields of 30%. According to Yang et al., microwaves were used to generate quantum dots using resorcinol, OPD, and hydrochloric acid, and a quantum yield of 62.8% was achieved. This was because the QDs had been doped with nitrogen, so there were many nitrogen atoms in the QDs. In addition, the dots contained few vacancies in the carbon skeleton, and the core consisted mainly of the sp^2^ domain. It was found that the reaction was positively affected by the presence of HCl as a “promoter”, resulting in bright fluorescence and a high yield of reaction products. Consequently, if this method is applied to waste biomass instead of chemical feedstock as a power plant raw material, it might be possible to reduce the ecological impact of its use [52].

## 5. Modification of QDs with Bioresource

Bioresources can be defined as organically produced raw materials that have been created by the activities of humans and animals and can, therefore, be used as natural, renewable resources that are derived from the material itself. Bioresources can be laboratory animals, plants, cells, genes, and micro-organisms used for research [53]. In addition to agricultural waste, carbonaceous solid waste is also a bioresource. There are various methods to dispose of agricultural wastes that are continuously generated from agricultural activities. Various industries, including mills, can use this waste as a fuel source for heating, power generation, and chemical processes. From these wastes, various industries, such as mills, can generate electricity, heat, and chemicals. GQDs derived from plant leaves do not require oxidizing or reducing agents or organic solvents, and the structures of these bioresources contain various functional groups [54]. Bioresources are used for various purposes and applications, as shown by agroforestry and aquaculture. The use of important bioresources for energy production is problematic due to the ineffectiveness of GQD. Synthesis received much attention due to their low cost, availability, and high carbon content as potential feedstocks for the synthesis of GQDs. Functionalization of GQDs, even when they are in pure form, occurs because of the constraints on their use. Functionalization is achieved either by forming composites with polymers [55], inorganic materials [56], organic molecules [57], etc., or through doping with heteroatoms [58]. The functionalized GQDs have enormous applications, such as drug delivery, bioimaging, batteries, sensors, etc. [59,60,61]. Recently, the synthesis of QDs from various bioresources has been influenced by the development of green and sustainable chemistry [62,63,64]. This is mainly because conventional synthesis methods involve lengthy synthesis processes under extreme temperature and pressure conditions. In addition, these synthesis methods use toxic reagents and require extensive purification procedures. In the synthesis of QDs, the use of bioresources led to inexpensive and simple methods. Therefore, the present study focuses on the synthesis of QDs from various bioresources, including micro-organisms, such as bacteria, fungi, yeast, and algae [62,65,66,67].

### 5.1. Microorganisms Derived QDs

**Figure 1:**
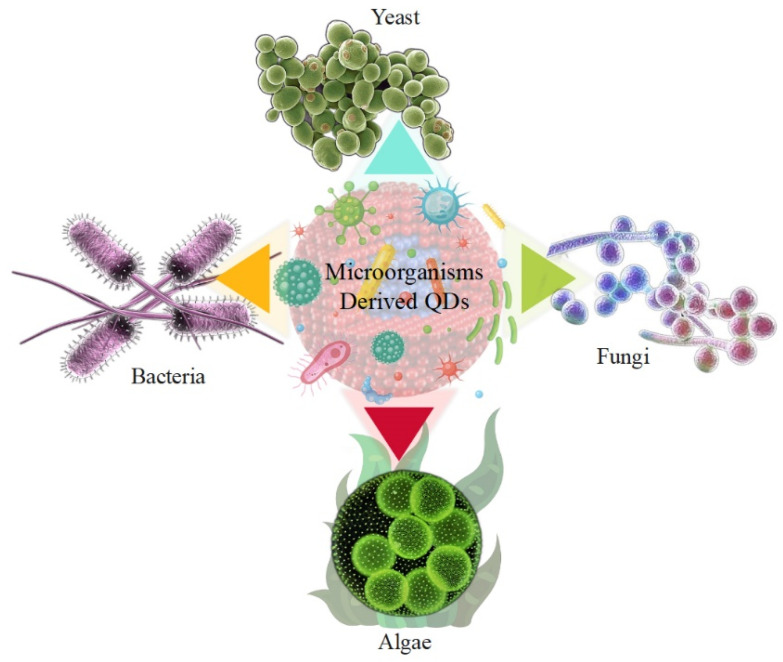
Micro-organisms derived QDs, such as bacteria, fungi, yeast, and algae.

As properties, such as biocompatibility, environmental friendliness, and feasibility, are considered in the implementation of synthesis methods, it is possible to propose the synthesis of QDs via biological pathways that are environmentally friendly, inexpensive, and low in toxicity, rather than using chemical synthesis methods for QDs. Micro-organisms are also excellent biological nanofactories for the synthesis of QDs. The biosynthesis of QDs has several major advantages that can be exploited, especially in energy production and storage. Micro-organisms have tremendous diversity, which gives them an inherent potential to mediate QDs. Bacteria, yeasts, and fungi are among the micro-organisms suggested to synthesize QDs. This is due to various factors, such as pH and temperature, their ability to grow under stressful environmental conditions, and their ease of cultivation [68,69]. Due to their high tolerance and self-compatibility in environments containing toxic metals, micro-organisms have the unique ability to promote QD synthesis by utilizing energy production and storage [70,71]. Microbial enzymes play a key role in the conversion of precursor metal ions into nanoparticles. Various biological sources are used for the biosynthesis of QDs, such as viruses [72], bacteria [73], fungi [74], agricultural and industrial wastes [75], plant extracts [76], and algae [77]. Figure 1 shows micro-organisms such as bacteria, fungi, yeasts, and algae that produce QD. Currently, the biological production of QDs by micro-organisms, such as yeasts, bacteria, and fungi, has attracted particular attention due to their ability to bioaccumulate and biotransform QDs, as shown in Table 2. The synthesis of QDs by fungi was also given attention because they were effective secretors of various biomolecules among the micro-organisms studied. The advantages of using fungi also include their cost-effectiveness and ease of biomass processing. However, the synthesis of QDs by bacteria offers advantages, since bacteria can be genetically engineered to express specific enzymes involved in the synthesis of QDs [78,79].

#### 5.1.1. Bacteria

**Figure 2:**
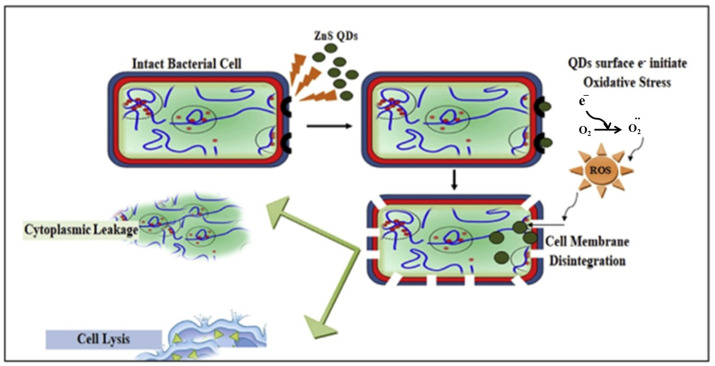
Cytotoxic effect of ZnS QDs on bacterial cells. Reprinted with permission from Ref. [108]. Copyright 2019 Elsevier.

Bacteria have great potential in energy-related fields because they can be produced on a large scale, have a diverse morphology, are biomineralizable, have unique electrochemical activity, and are inexpensive. In addition, they are considered to be the most widely distributed living organisms and provide the largest contribution to the material cycle in the natural environment [103,104,105]. Economic production, stability, water solubility, and a stable structure are among the advantages of using bacteria for the biosynthesis of QDs compared to conventional methods. Another advantage of using bacteria for biosynthesis is the relatively low cytotoxicity of QDs. For the production of QDs of uniform size with potential antibacterial properties, the use of bacteria is suitable as an environmentally friendly, cost-effective, and simple method. The QDs prepared using bacteria showed excellent salt stability due to the morphological changes in the QDs caused by the bacteria. There is no doubt that QDs offer many exciting and indispensable prospects in the field of energy conversion. This is due to their diverse and strong physicochemical properties and advantageous features, such as quantum confinement effects and an abundance of surface defects. Doping with different heteroatoms cannot only enhance the electronic conductivity, but also introduce multiple active sites, increase the number of defects, and improve the chemical adsorption ability, which can improve the reaction activities and electrochemical kinetics in energy storage and conversion devices [10,106,107]. The bioinspired synthesis of ZnS QDs from *Aspergillus* sp. was investigated by Jacob et al. The results showed that biogenic ZnS QDs exhibited significant antimicrobial activity comparable to that of standard antibiotics after screening their antibacterial activity against common pathogenic bacteria. Factors responsible for the excellent bioactivity include the ability of ZnS QDs to disrupt the bacterial cell membrane, oxidative damage caused by ROS leading to bacterial cell lysis, and leakage of cytoplasmic contents (Figure 2) [108]. Table 3 shows the properties of QDs and the characterization tools for QDs biosynthesized from bacteria.

#### 5.1.2. Fungi

**Figure 3:**
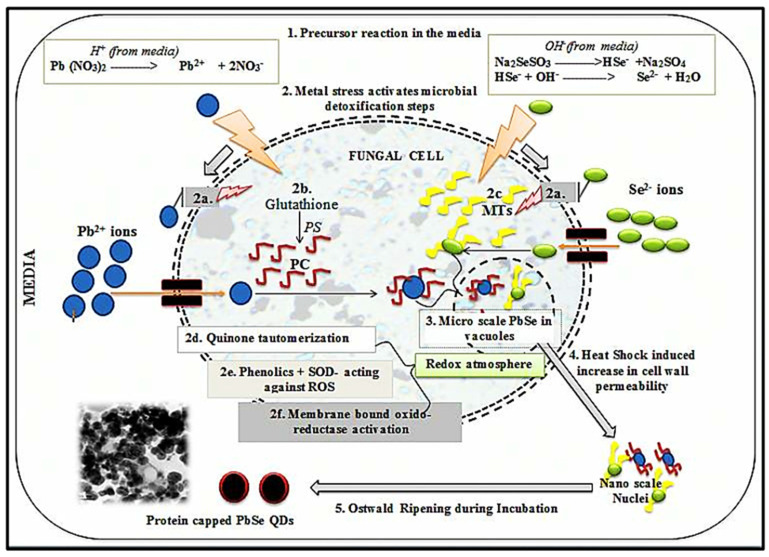
Mechanistic pathway of PbSe QDs biosynthesis from fungus. Reprinted with permission from Ref. [125]. Copyright 2017 Elsevier.

Heterotrophic micro-organisms with a nucleus and a cell wall are called fungi. In recent decades, fungi have attracted much attention in energy−related fields due to their high production, diversity, and rapid reproduction [118,119]. Fungi include micro-organisms, such as yeasts [120], and multicellular organisms, such as molds [121,122]. Since nano−dimensional particles with better monodispersity can be obtained by using fungi, fungal-mediated synthesis is more advantageous than bacterial synthesis. Therefore, the use of fungi is one of the most stable and effective methods for the synthesis of CdS QDs with high biocompatibility and excellent optical properties for the generation and storage of light. The use of fungi in the preparation of QDs also increases their fluorescence properties. In addition, the production of QDs with fungi has increased photocatalytic activity, which has led to greater degradation of pollutants and the degradation of biosensors used in the generation and storage of energy. Fungi and QDs can demonstrate their potential in energy-related fields, such as supercapacitors, lithium-ion batteries, and solar cells [123,124]. A fundamental mechanistic aspect of the biosynthesis of QDs from fungi was investigated by Jacob et al. As shown in Figure 3, the possible mechanism of biosynthesis of PbSe QDs from *Aspergillus tereus* was revealed by them. Detoxification of metals by biological molecules occurs through a series of events, such as the induction of high circulation of organic acids, the induction of proteins, and the stimulation of antioxidant enzymes, including the mechanism of fungal biosynthesis that emerged from their study. This process includes the following steps: (i) First, in the production medium of metal ions (Pb^2+^ and Se^2−^), the precursors (Pb(NO_3_)_2_ and Na_2_SeSO_3_) undergo redox reactions. (ii) Detoxification is initiated by several processes: (a) reversible combination of level functional groups (e.g., thiols, oxalic acids) with metal ions; (b) conversion of glutathione to phytochelatins (PC) due to activation of phytochelatin synthase (PS) and also binding with metal ions, eventually leading to their transport by ATP-binding cassette membrane transfer proteins into the cell vacuole is due to metal stress; (c) binding of metallothioneins to metal ions occurs in a similar manner; (d) redox reactions occur by tautomerization of quinine; microscopic PbSe is formed by the activity of superoxide dismutase (SOD); and (f) stimulation of other oxidoreductases to generate a redox environment. (iii) The manufacturing process of PbSe QDs occurs through the creation of a redox atmosphere and the involvement of glutathione/metallothionein. (iv) Thermal shock increases the permeability of the cell walls so that their contents are released into the environment. (v) Finally, nuclei form the QD−capped protein culture medium due to Ostwald maturation [125,126].

#### 5.1.3. Yeast

**Figure 4:**
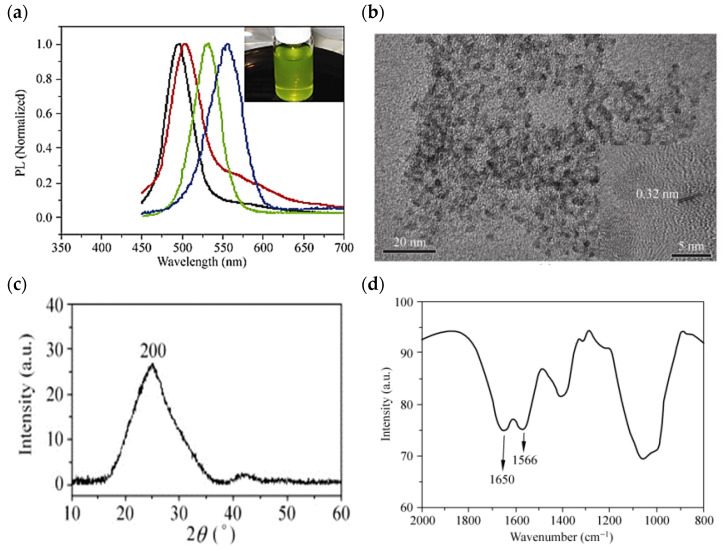
(**a**) Spectra of the biosynthesized CdTe QDs, which were taken from the reaction flask and centrifuged to remove the yeast cells after incubation at 35 °C in the open air for 1 day (black line), 2 days (red line), 4 days (green line), and 8 days (blue line). The inset in (**a**) is a photograph of the as-biosynthesized CdTe QDs under ambient room light. (**b**) TEM image (**c**) XRD pattern of the CdTe QDs extracellularly bio-grown with the yeast cells at 35 °C for 8 days (**d**) The FT–IR spectrum of the biosynthesized CdTe QDs incubated with yeast cells at 35 °C for 8 days. Reprinted from Ref. [132].

Yeast is a unicellular fungus that belongs to the facultative anaerobes and can live in aerobic and anaerobic environments [118,127,128]. Yeast cell colonies are much thicker and larger than bacteria [129,130,131]. In addition, yeasts are widely used in industry for energy production and storage. CdTe QDs are among the most important QDs considered for energy storage applications due to their unique properties, which include high quantum efficiency, control, and narrow emission spectra. Bao et al. investigated CdTe QDs biosynthesized with yeast cells with tunable fluorescence emission spectra. They showed the size-dependent emission spectra of the as-prepared CdTe QDs at 490–560 nm, as shown in Figure 4a. They also identified the dispersion of CdTe QDs with a diameter of 2.0–3.6 nm at TEM, as shown in Figure 4b. In the XRD pattern at 2θ ~ 26.7 °C, the diffraction peak of the biosynthesized CdTe QDs is cubic and corresponds to the (200) reflection in Figure 4c. By FTIR spectroscopy, it is possible to identify the possible ligands coating the fabricated CdTe QDs. Two absorption peaks corresponding to the amide I and II functional groups were found at 1650 and 1566 cm^−1^, respectively, as shown in Figure 4d. The results show that the microbially prepared CdTe QDs exhibit exceptional biocompatibility and stability, and also have a high quantum efficiency of ~33% [132].

#### 5.1.4. Algae

**Figure 5:**
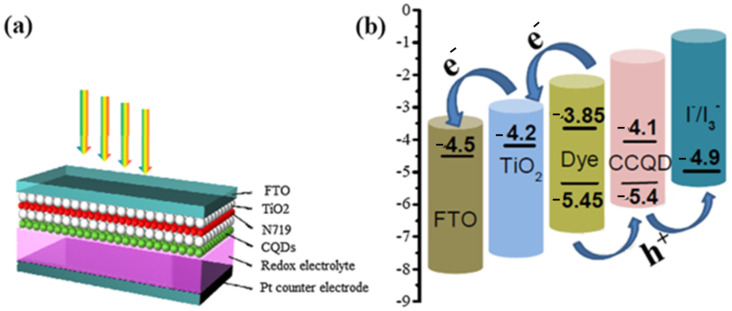
(**a**) Schematic of solar cell structure with the help of CQDs as light harvester (**b**) charge transfer and energy level distribution of solar cell photoanode. Reprinted with permission from Ref. [135]. Copyright 2019 Elsevier.

Recently, algal-mediated synthesis of QDs for various energy generation and storage applications has attracted attention due to its importance in the development of biocompatible and highly fluorescent QDs. Since one of the environmental problems is caused by algal biomass, the use of algae for the biosynthesis of QDs helps not only as a bioresource for synthesis but also for environmental remediation. The results of using algae as precursors include the remarkable properties of QDs, including excellent blue luminescence in UV light, ionic strength, commendable stability, pH, exceptional insensitivity to photobleaching, and high-water dispersion. Another result of using algae is excellent light transmission. QDs synthesized by this method showed efficient optical absorption and fluorescence. The bioresource of microalgae is a sustainable, renewable, and abundant source that provides a cost-effective and easy way to synthesize CQDs for energy generation and storage [133,134]. A mesoscopic solar cell system was developed using CQDs synthesized from Gelidium amansii powder, a species of red algae, to serve as light collectors. According to Dou et al., the CQDs were able to harvest light effectively. A mesoscopic TiO_2_ photoanode, in which CQDs containing the dye N719 were used, served as the photocell. The counter electrode, created from FTO glass with a Pt carrier, was used as the redox electrolyte for the solar cell. In addition, CQDs, N719, and TiO_2_ with a FTO glass support were used for the counter electrode (Figure 5a). Since the HOMO edge of the CQDs agrees well with the value of the N719 dye, and as evidenced by the redox potential of the I*/I3* system, extraction of holes from the N719 dye into the electrolyte occurs, leading to an improvement in the separation of electrons and holes (Figure 5b) [135].

### 5.2. Bio-Wastes Derived QDs

Animals and plants engage in a variety of activities that result in the generation of biowaste, which can be divided into several categories. As a renewable energy resource, biowaste is converted into GQDs without the addition of chemicals [136]. Tade et al. converted industrial waste and biowaste into GQDs without adding chemicals. A study on carbonaceous bioprecursors, such as amino acids and carbohydrates, was carried out to understand their processes [137]. Based on the work of Mohan et al., it was found that the synthesis of GQDs can be successfully carried out using sugarcane bagasse (SB) as the feedstock for the process. This experiment was carried out at room temperature, and the starting material was carbonized during the experiment. To perform the synthesis of sugarcane bagasse, 2 g of bagasse was mixed with 2 g of NaNO_3_ and 26 mL of H_2_SO_4_. This product was then mixed with 6 g KMnO_4_ and oxidized for 48 h with constant stirring. It was then mixed again with 6 g KMnO_4_. After the reaction was complete, I added 500 mL of distilled water and 5 mL of H_2_O_2_ to the mixture. The resulting sample was then diluted to ensure that no traces of acid remained in the sample [138].

#### 5.2.1. Lignin

**Figure 6:**
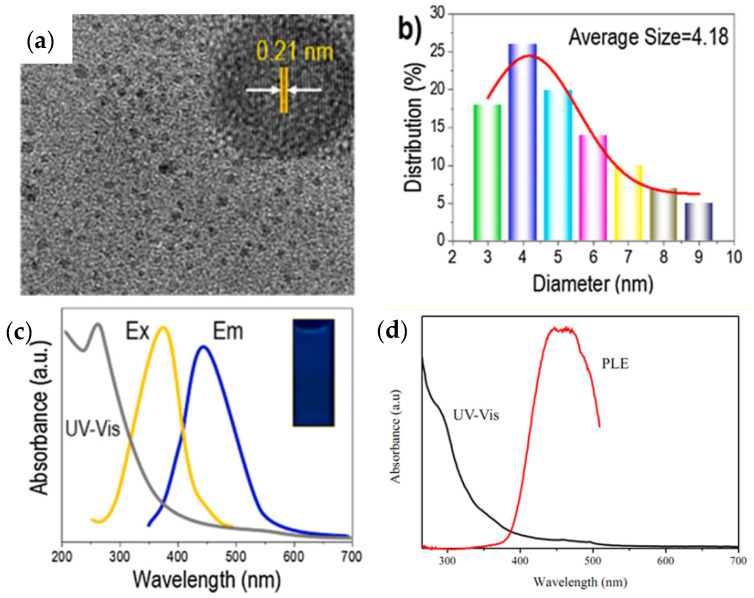
Structural profile and performance of CQD assay from lignin. (**a**) TEM images (scale bar 50 nm), (**b**) high-resolution XPS spectra of graphene, particle size distribution, (**c**) UV-vis absorption spectrum, emission spectrum under maximum excitation at 360 nm, and excitation spectrum observed at maximum emission wavelength of 440 nm. Reprinted with permission from Ref. [146]. Copyright 2021 Elsevier. (**d**) UV–Vis and PL excitation (PLE) spectra of C dots. Reprinted with permission from Ref. [144]. Copyright 2019 Elsevier.

Lignin-based QDs materials are usually environmentally friendly and low cost, and are widely used in energy storage, the environment, electronic devices, and other fields [139,140]. It should be noted that lignin is attractive for the fabrication of CQDs due to its high graphene content and aromatic phenylpropane structure, which has the great advantage of resembling the graphene framework of CQDs. To obtain very bright CQDs with stable fluorescence emission and water solubility, simple hydrothermal processing of LPC can also be used. Transmission electron microscopy (TEM) can be used to determine the morphology of lignin-based CQDs (see Figure 6a). The TEM image also shows good dispersion of the quasi-spherical CQDs with no obvious aggregation. One of the reasons for the particles having a highly crystalline graphene structure is the presence of (100) graphitic carbon layers (0.21 nm lattice spacing) in the high-resolution TEM image, the TEM image shows stripes with a spacing of 0.21 nm [141]. The uniform distribution responsible for the photoluminescence and confinement of CQDs can be confirmed by the distribution of CQDs in the range of 2 to 9 nm with an average diameter of 4.18 nm (Figure 6b) [142]. Figure 6c shows the location of the strong absorption peak at approximately 265 nm in the sp^2^-hybridized graphene core, which is due to the π-π* transition of the conjugated C=C/C=O bonds [143,144]. The color change of the lignin-based CQD solution under a UV lamp with a wavelength of 360 nm from pale yellow in natural light to light blue fluorescence is shown in the inset photograph in Figure 6c. Additionally, in Figure 6d, the strongest absorption of CQDs by PL excitation, which is similar to the documented PL excitation analysis of carbon dots (Cdots), was shown in the range of 400–460 nm [144]. The CQDs showed an excitation peak approximately 449 nm, which could be due to the graphene core, surface structure and specific diameter [145].

#### 5.2.2. Wood Charcoal

There are many methods of making charcoal, but the most common is to heat wood at 400 degrees Celsius in an oxygen-deficient environment, since charcoal is a pure form of carbon. Pyrolysis is a method of converting wood into charcoal and is one of the most effective methods. As an abundant, highly flammable energy source, charcoal, a carbonaceous substance, is readily available in large quantities and is an abundant carbon source [147]. GQDs were synthesized in a two-electrode system by electrochemical oxidation of charcoal as described by Nirala et al. In this experiment, a solution of 0.01 M ammonium persulfate was used as electrolyte and an electrode potential of 5 volts was used for oxidation, with a current of 100–200 mA/cm^2^ during oxidation. The free rotation that occurs during the anodic oxidation of water and giant graphene sheets allowed the giant graphene sheets to be oxidized to GQDs. The assembly was centrifuged in a centrifuge at 10,000 rpm for 20 min. E-GQDs were also prepared by dialyzing a mixture for a period of three days before analysis [148].

#### 5.2.3. Coffee Grounds

It has been observed that as coffee consumption has increased, so has the production of ground coffee, which is rich in carbohydrates, proteins, caffeine, tannins, and pectins, all of which have been shown to be important for human health. In addition to the primary composition of ground coffee, carbon is also a component of its primary composition. Carbon can be used in a variety of applications, such as wastewater treatment, biomedical waste treatment, and other industrial processes [149].

Recently, a study was published by Wang et al. using coffee grounds to synthesize GQDs. For this purpose, 0.1 g of highly sterile and dried coffee grounds was mixed with one ml of hydrazine hydrate. It was sonicated with 10 mL of water and 0.1 gram of coffee grounds for 30 min. The mixture was then kept at 150–200 °C for approximately six–ten hours. After filtering off the solution containing the water-soluble GQDs, small molecules were removed from the solution by dialysis for two days. The quantum yield was reported to be 33%. By using these GQDs for bioimaging, ion detection, and environmental analysis, we can produce blue fluorescent GQDs that emit blue fluorescence at 470 nm [150].

## 6. Applications of Energy Generation and Storage Devices by Bioresource-Derived QDs

### 6.1. Integrated Devices for Energy Harvesting and Storage

**Figure 7:**
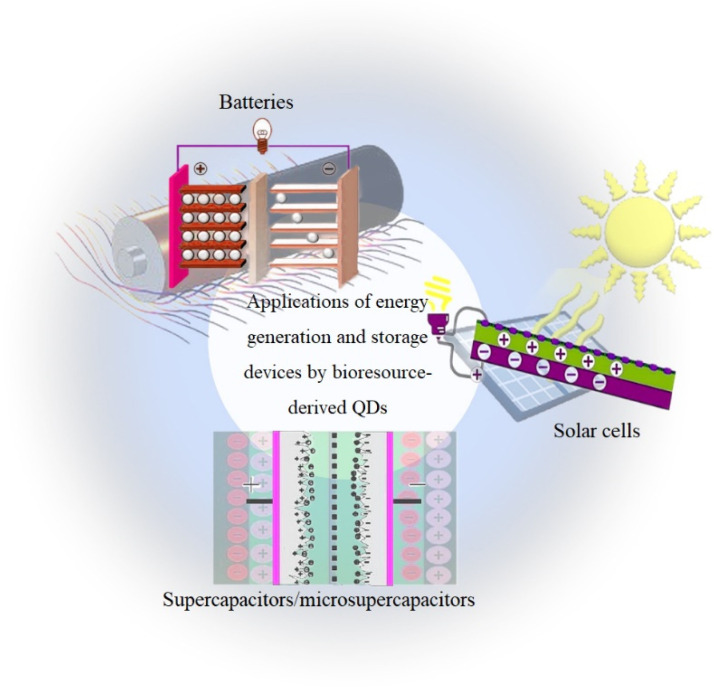
Applications of energy generation and storage devices by bioresource-derived QDs.

Integrated energy harvesting and storage devices, such as SCs/MSCs, lithium-ion batteries (LIBs), and solar cells, play an important role in daily life because they can replace conventional fossil fuel energy (Figure 7). Nevertheless, these isolated devices cannot provide enough energy for long-term operation and constantly changing work situations, and have limited power and/or exclusive use. This shows that the development of good automated systems is necessary to meet the increasing energy demand for long-term use in different environmental scenarios. An effective way to achieve an energy system with high density, small size, and high reliability is to develop an integrated energy package and a combination of energy harvesting and storage [151]. One of the most important energy-related technologies that can replace the battery or at least extend its lifetime is energy harvesting and storage devices [152]. The use of toxic chemical reagents, high temperatures, high time consumption, and synthetic steps are among the drawbacks of many traditional methods of bioresource-derived QD synthesis that limit the application of these syntheses. Therefore, recently, new bioresource-based methods, such as those using micro-organisms (bacteria, fungi, and yeasts), lignin, and cellulose, are being used as graphene sources for the synthesis of bioresource-derived QDs. Considering the several advantages of bioresource-derived QDs, including good recyclability, convenient synthesis, colorful PL, excellent biocompatibility, and low cost, scientists have been inspired to develop novel materials with low environmental impact and new applications, such as supercapacitors/microsupercapacitors, batteries, and solar cells [153,154,155]. In recent years, properties, such as enhanced absorption of UV light, ease of functionalization, and improved efficiency of solar cells have been used to improve the performance of solar cells with QDs [156]. A QD is a type of nanoparticle that can be used as a mobile material to improve the performance of batteries by introducing it into their electrolytes to improve their electrical properties [157].

### 6.2. Supercapacitors/Microsupercapacitors

**Figure 8:**
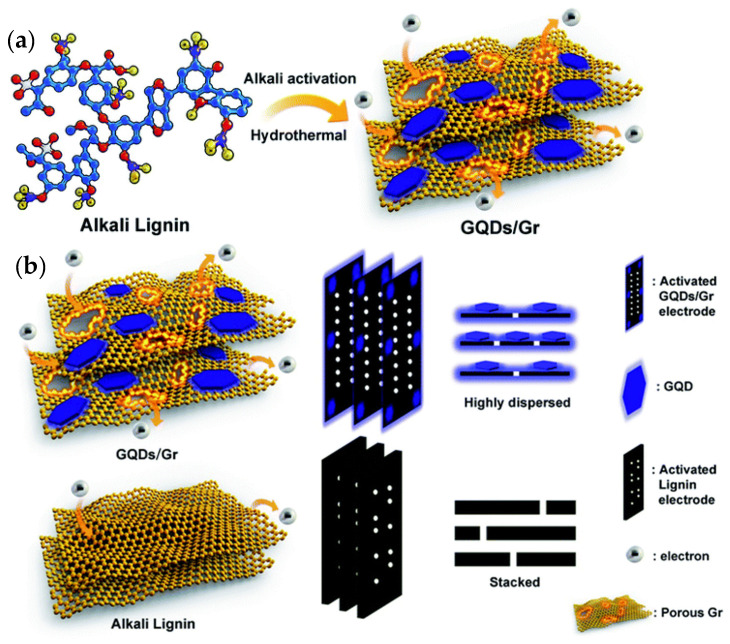
(**a**) Design of a 0D/2D GQD/Gr-converted all-lignin heterogeneous graft by a low-cost, facile, and scalable alkaline-activated hydrothermal method for high-speed, specially enhanced supercapacitors. (**b**) Schematic illustration of more electron storage and efficient emission towards the GQD/Gr electrode through the GQD/Gr lignin electrode and alkali. Reprinted from Ref. [160].

SCs/MSCs as energy storage devices have attracted more attention due to their unique advantages, such as short charge/discharge times, small size, long lifetimes, and high-power densities. SCs/MSCs can be divided into two types of capacitors based on different charge storage mechanisms: quasi-capacitors and electrochemical double-layer capacitors. In general, SCs/MSCs with GQDs, whose mechanism is charge absorption at the electrode level as the electrode material, belong to the electrochemical double-layer type. Xue et al. investigated the electrochemical deposition of GQDs on a gold finger electrode. The results showed that the MSCs obtained after 5000 cycles have an operating speed of up to 1000 V/s, a specific capacitance of 534.7 mF/cm, and a specific capacitance of 97.8% compared to their initial specific capacitance [158]. A flexible, transparent MSC with highly bendable properties and a specific capacitance and high transmittance at 550 nm wavelength of 9.09 mF cm^−2^ and 92.97%, respectively, was developed by Lee et al. using chelated graphene and GQDs. It retains approximately 100% of its initial specific capacitance with respect to bending after 10,000 continuous cycles (long cycle stability can be maintained) [159]. Most lignin-based supercapacitors generally exhibit slow diffusion kinetics and lower electrochemical performance with low capacitance due to their low conductivity, uncontrolled morphology, and poor interfacial compatibility. Ding et al. investigated the simultaneous rapid charging and increase in specific capacitance with GQDs and heterogeneous graphene sheets (GQD/Gr) prepared entirely from lignin. The in situ growth of GQDs on graphene is possible due to the conversion of lignin into GQDs and subsequent deposition on graphene, which provides good surface compatibility with GQD/Gr heterogeneity. They found that the GQD/Gr heterojunction has a high specific capacitance of 404.6 F g^−1^ and a short charge time constant (τ_0_) of 0.3 s, which is 2.5 times longer and 7.5 times faster than the unmodified lignin electrode with 162 and 2.3 s, respectively. The results show that it is possible to overcome the critical barriers for a lignin-based supercapacitor with superior electrochemical performance by creating a discontinuous 0D/2D GQD/Gr system. It also paves the way for the conversion of high-quality industrial lignin into highly efficient, scalable, and cost-effective electrochemical energy devices (Figure 8) [160]. The various features and functions of GQDs in MSCs and SCs are shown in Table 4.

### 6.3. Batteries

The use of LIB as a power source and in electric and hybrid vehicles as an intelligent energy storage system is becoming increasingly popular. Due to their lightweight design, cost-effectiveness, energy efficiency, and long life, the commercial demand for these batteries is increasing significantly. The charge-discharge process can rapidly degrade the capacity of many LIB electrode materials, but many of them also exhibit poor rate performance due to self-aggregation, uncontrolled volume expansion, dissolution, formation of solid electrolyte interfaces across the electrodes, and a rapid increase in charge transfer resistance during cycling [166,167,168]. Although electrode materials for LIBs are widely used, they have a number of drawbacks. These include the fact that the electrode materials are not coulombically efficient, electrolyte depletion, and safety concerns. Recently, the use of QDs incorporated into LIB electrode materials has proliferated to address the issues associated with these materials. In order to improve the efficiency of the next generation of LIBs, changes are being made to the electrode materials in terms of surface finish and internal structures. Compared to other types of electrode materials, QDs possess a number of advantages, including increased conductivity, an increased number of active surfaces on the electrodes, and improved electrode surfaces and wettability, all of which are important aspects of electrode material performance. In addition, they could also help control the volume expansion of electrode materials and control the voltage drop during charging and discharging. In addition, anode materials with a large surface area, morphology, and high dimensional stability have also been explored as anode materials because they have a variety of properties that make them suitable for use in anode applications, such as graphene and carbon nanotubes. By weakening the lattice disorder in these materials, they cannot only be strengthened but can be used more effectively as anodes in LIBs by enlarging the defects, improving the order in the lattice, and creating pores. Graphene and carbon nanotubes (CNTs) accumulate defects over time as a result of interactions between van der Waals forces and high surface energies. To understand the extent of the problem of accumulated defects over time, it is particularly important to understand how these defects accumulate over a short period of time. Due to the high conductivity of graphene and carbon nanotubes, composites can be created that combine these highly conductive graphene and carbon nanotubes by duplicating graphene and carbon nanotubes and then combining them. The functional groups (-OH and COO-) on graphene quantum dots create a negative charge on the surface of the CNTs. This prevents the CNTs from clustering together, as molecules with the same charge repel each other. In addition, these functionalized CNTs improve the storage capacity of Li-ion batteries. The combined performance of these hybrid graphenes and carbon nanotubes was greater than the performance of the individual elements. The addition of these active sites and the reduction in mechanical stress also led to an increase in the number of active sites. In addition, a reduction in mechanical stress is also caused by the volume changes associated with the charge/discharge processes [169,170,171].

**Figure 9:**
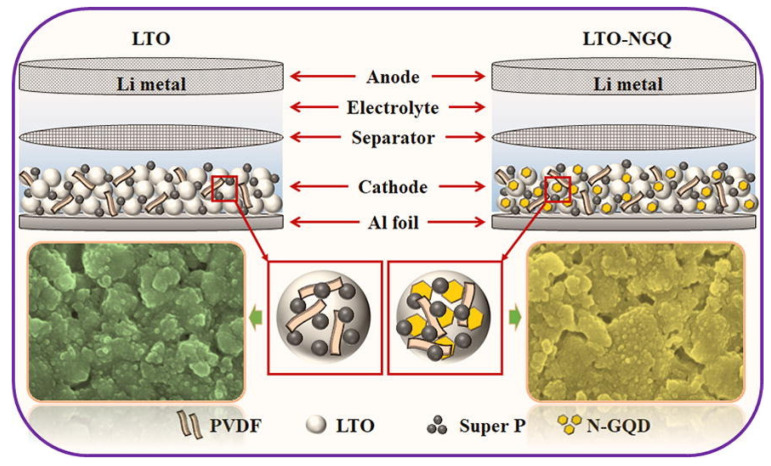
The electrochemical performance of LTO-NGQ20 with rate capability from 0.2 °C to 50 °C and long cycling performance electrodes. Reprinted with permission from Ref. [176]. Copyright 2019 Elsevier.

GQDs are used in lithium-ion batteries due to their unique structure and high conductivity, low density, high hardness, and high tensile strength [172,173,174]. The bioresource-derived GQDs improve the electrochemical performance in Li^+^ or Na^+^ batteries, increase the electron transfer and Li^+^/Na^+^ diffusion rates, and decrease the volume expansion. Electrochemical performance can be improved by mixing bioresource-derived GQDs with an active material as an auxiliary material or by using bioresource-derived GQDs as an anode material. VO_x_ is considered a promising electrode material for Li-ion batteries because it offers high capacity, low cost, and abundant resources. They have fabricated a number of VO_X_ nanostructures to use as LIB cathodes, and so far, we have been able to report on their fabrication. Nevertheless, most of these materials tend to drop capacitance rapidly and perform poorly at high speeds, while resistance increases rapidly during cycling, suggesting that they are not suitable for use in extreme conditions [157,175]. A new LTO/N-GQD/superhierarchical anode material was investigated for LIB by Khan et al. The results showed that LTO with N-GQD not only has better electrical properties, but also increases the specific capacitance by 23% compared to pure LTO (Li_4_Ti_5_O_12_); therefore, the discharge capacity for more than 200 cycles is approximately 170 mAh·g^−1^ at 20 °C, as shown in Figure 9 [176].

### 6.4. Solar Cells

**Figure 10:**
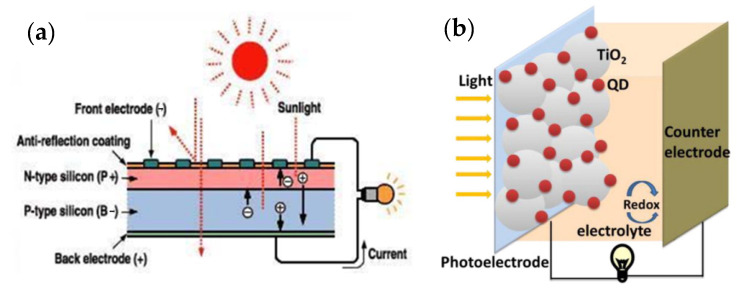
(**a**) Schematic diagram of a solar cell. Reprinted from [192]. (**b**) Schematic design of a solar cell sensitized to QDs. Reprinted from Ref. [193].

A solar cell or photovoltaic cell is a solid-state electronic element that converts a portion of received solar energy directly into electricity. The operating mechanism of the solar cell is that by generating a photon and absorbing it, an electron-hole pair is created. By connecting two p-type and n-type semiconductors, electrons are transferred from the n-type region to the p-type region, and holes are transferred from the p-type region to the n-type region. The positive and negative ions created by the electron-hole transfer generate an electric field that becomes stronger as more electrons and holes are transferred until the formal levels of the two regions equalize and an electric field is created. Upon solar irradiation and photon absorption (light packets whose energy is higher than the energy of the semiconductor gap), the generated electron-hole pair penetrates the electron donor-acceptor interface. Then the electron-hole pair is separated at the interface and generates electrons and holes. The electric field drives the electrons into the n-region and the holes into the p-region. In this way, a potential difference is created due to the negative charge density in the n-region and the positive charge density in the p-region. Then the charge transfer to the cathode and anode takes place. When the cathode and anode are connected with a wire, the excess electrons in the n-region migrate through the wire to the p-region, and a short-circuit current is generated. Figure 10a shows a schematic diagram of a solar cell. Nowadays, silicon solar cells are used on an experimental scale, but since the silicon wafers must have a high degree of purity, the manufacturing cost is very high, which makes it difficult to use this type of solar cell. Different types of polymers, pigments, and perovskite solar cells have been produced, which have reasonable energy conversion efficiencies and lower weight and cost. A solar cell consists of three parts: photoanode, cathode (usually metals, such as aluminum, silver, and calcium), and active layer. Generally, indium oxide is used as a photoanode. This compound has a suitable bandgap, high transparency and electrical conductivity, and has a suitable working function. Due to the limited indium sources, expensive fabrication methods, instability in acidic environments, and brittleness of electrodes, the attention of scientists has been drawn to the use of materials with better performance, such as photoanodes or electrodes [177,178,179,180]. Bioresource-derived QDs have other advantages due to their special properties, such as lightweight and low density, high electrical conductivity (with zero bandgap and good mobility for electrons and holes), and flexibility, which include availability, affordability, sustainability, and an ecofriendly nature; they also have disadvantages, including scaling problems due to uneven heating and a wide size distribution of solar cells [181,182,183]. Since the mass production of bioresource-derived QDs, intensive research on bioresource-derived QDs has been carried out in various industrial fields, especially in solar cells [184]. Bioresource-derived QDs solar cells are developed based on Gratzel or pigment-sensitive solar cells, but they use low-bandgap semiconductor compounds that can be used to fabricate bioresource-derived QDs, such as PbS, Sb_2_S_3_, CdSe, and CdS. In these devices, QDs derived from bioresources are used instead of light-absorbing organic or metal-organic pigments. Changes in the size of QDs derived from bioresources lead to changes in the intensity of the wavelength of light absorbed in these cells [185]. In solar cells sensitized with bioresource-derived QDs, the mesoporous TiO_2_ layer forms the main skeleton of the cell, just as it does in cells sensitized with pigments, and the deposition of bioresource-derived QDs on the TiO_2_ layer leads to light sensitivity in these structures. The quantum particles can be deposited on the TiO_2_ layer by various methods, such as chemical bath deposition, electrophoretic deposition, or successive ionic layer adsorption and reaction (SILAR). Finally, the cycle is closed with a reducing/oxidizing (redox) couple, either solid or liquid. The efficiency of solar cells sensitized with GQDs in liquid crystal cells and solid-state cells has reached more than 5% [186,187,188]. Bioresource-derived GQDs are widely used in high-performance photovoltaic devices, including organic-inorganic hybrid solar cells [189] and perovskite solar cells [190,191]. Figure 10b shows a schematic representation of the structure of a solar cell sensitized with bioresource-derived QDs.

#### 6.4.1. Organic and Inorganic Hybrid Solar Cells

**Figure 11:**
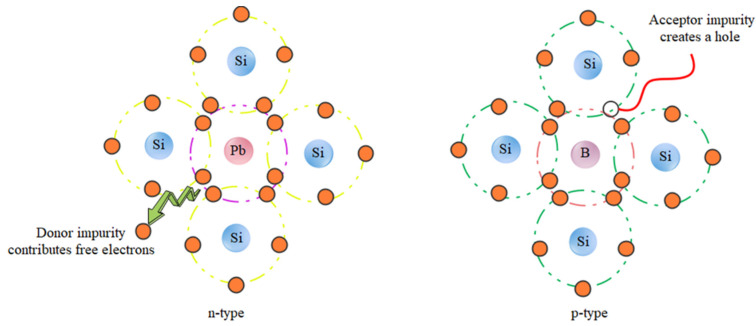
Mechanism diagram of the semiconductor physics-related content.

Silicon crystal cells are the first generation of solar cells. This type of solar cell, with an efficiency of approximately 22%, dominates the world market. However, despite the high efficiency and nontoxicity of the materials, there are limitations in the production of inorganic cells, namely: high production costs, inflexibility, and fragility of this type of cell. Inorganic silicon cells consist of two semiconductors (n) and an acceptor (p). When sunlight shines on the p-n junction (junction between two semiconducting and accepting materials), photons with energy greater than the bandgap energy of the semiconductor create electron-hole pairs, and the pairs formed in the zero region have a high chance of being separated by the internal field before recombination (Figure 11) [194,195]. Organic solar cells consist of conducting polymers or other organic conductors as electron transfer materials. The basis of their work is similar to that of the inorganic solar cell. Polymers with different LUMO (lowest unoccupied molecular orbital) and HUMO (highest occupied molecular orbital) levels are bonded together. In general, the process of energy generation in an organic solar cell is divided into several stages, including the absorption of light and formation of excitons, emission of excitons in the direction of transition, dissociation of excitons into charge carriers, and transfer of charge carriers to the electrodes and their collection [196]. Therefore, organic/inorganic hybrid solar cells are of increasing interest in fabricating low-cost organic photovoltaics (OPVs) and to achieve other advantages, such as tuning of the absorption spectrum, with inorganic components. This is because hybrid solar cells have the potential to achieve high power conversion efficiency (PCE), but it is currently low. To increase the power conversion efficiency, mineral materials are used as electron acceptors in hybrid solar cells. In particular, the electronic structure is very important for the device’s performance [197]. Photon down-conversion is a property of bioresource-derived GQDs that enables the absorption of photons in shorter wavelength ranges for optical applications. Tsai et al. used and investigated GQDs as down-conversion materials for heterogeneous silicon solar cells. The short-circuit current in different layers of solar cells increased from 35.31 to 37.47 mA·cm^2^ by adding GQDs in an amount of 0.3 wt% [198]. Additionally, for hybrid solar cells, a yield of 13.22% was obtained using GQDs [199]. A high detection rate of 8 × 1011 Jones without applying bias voltage can be achieved by introducing GQD as an additive for photon down-conversion in PEDOT:PSS through the hybrid device [200].

#### 6.4.2. Perovskite Solar Cells

**Figure 12:**
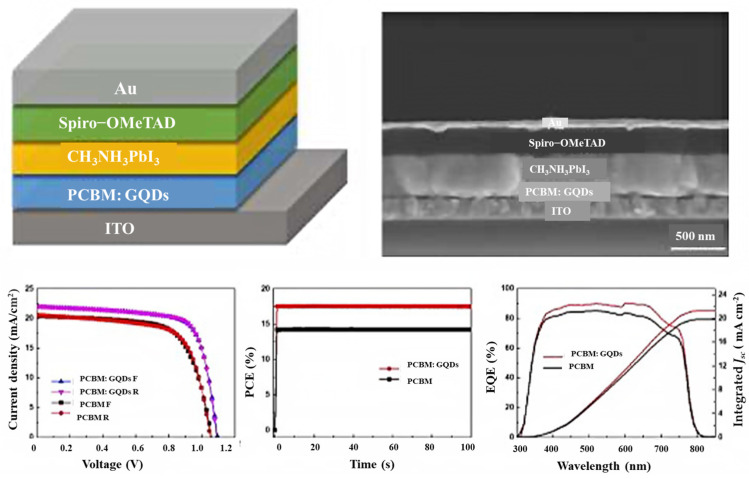
PSCs with a GQD-doped PCBM electron transport layer. Reprinted with permission from Ref. [210]. Copyright 2017 Elsevier.

The latest generation of solar cells that has attracted the attention of scientists around the world since 2014 is perovskite solar cells. This group of solar cells consists of perovskite compounds, which are usually organic mineral-lead hybrids or tin halide-based materials. Perovskite materials have two very important advantages: they are cheap to produce and easy to process. The low manufacturing price and high efficiency of this group of solar cells will lead to a large market for these solar cells by 2017 [201,202,203,204,205,206,207]. Bioresource-derived GQDs and graphene microlayers are among the advanced nanomaterials that have good chemical stability and tunable band gaps. Moreover, bioresource-derived GQDs act as excellent electron acceptors and donors in photoextraction [208]. To obtain efficient planar perovskite solar cells, Zhang’s group investigated GQD-modified TiO_2_ films of different sizes in 2017. They also found that GQDs can decrease the effective electron transport at the perovskite/TiO_2_ interface and the contact resistance. This reduces the series resistance and increases the combined resistance of the device, which in turn improves the fill factor and open circuit voltage (VOC). The results show that GQDs increase all PV parameters by 19.11% to achieve the best PCE [209]. To improve the performance of planar perovskite solar cells (PSCs) using GQDs, Yang et al. reported a powerful method, such as a planar phenyl-C61 butyric acid methyl ester electrotron transport layer (PCBM ETL) with additional forward PSCs, which dramatically increases the power conversion efficiency (PCE) of PSCs while improving the optical stability of the devices. While the PCBM GQD device maintained a maximum of 80% under continuous full-spectrum solar irradiation for 300 h, their study showed that the PCE increased from 14.68 to 17.56 (Figure 12). The results showed that the conductivity under GQD doping conditions is an order of magnitude higher compared to pure PCBM [210]. Therefore, the improvement of electron transfer in the perovskite layer, the deactivation of large parts of the grain boundaries, the reduction in charge recombination, and the elimination of electron traps are among the advantages of introducing GQDs into the perovskite thin films.

## 7. Conclusions and Outlook

One of the biggest challenges of bioresource-functionalized QDs for energy generation and storage is the low production yield (or unreported). While the analysis is performed in solution, the yield can be such that it is impossible to separate the dots in solid form. However, in addition to reporting yield or lack thereof, researchers should always provide a reason for low yield (even if the yield is so low that it is impossible to quantify). Knowing these challenges helps researchers who need more effort to improve the yield of solid-dot synthesis methods. Additionally, to increase the viability of QDs derived from bioresources in applications related to energy generation and storage, increasing yield plays a significant role. Quality is as important and necessary as quantity. Optimization for efficient and large-scale production methods is important for the synthesis of high-quality QDs with small, uniform sizes, and high quantum yields. Size uniformity means that the emission properties are uniform and have a narrower range; meanwhile, the high quantum efficiency increases the brightness of the dots. The smaller size increases the surface area for functionality and allows them to be used in a wider range of applications, especially in energy generation and storage. These properties are much better for applications, especially in microcapacitors/supercapacitors, batteries, and solar cells. Not fully explaining the optical properties is another challenge that needs to be solved. Therefore, more research should be conducted to fully understand the mechanisms behind PL in different types of QD and how to control them, such as doping, effect size, and surface defects. Researchers should periodically sample during QD synthesis to report on the mechanisms. Finally, the analysis of these samples can be used to suggest the appropriate mechanism because it clarifies what happens during the synthesis. Additionally, more research is needed to describe the effect of changing the synthesis parameters on the physical and optical properties of QDs. To further describe the relationship between physical and optical properties, reports on a wide range of dot properties with synthesis conditions can be used. The synthesis of dots that have better optical properties and that can be tailored to their specific applications is possible through a better understanding of the link between physical properties and optical properties, as well as through a better understanding of PL mechanisms. Bioresource-derived QDs are currently moving toward green synthesis consideration, and some syntheses are already green-labeled. Therefore, it is expected that syntheses will use green principles as much as possible in the future. Additionally, syntheses should be efficient in terms of reducing reaction waste, energy, and atomic economy. This type of synthesis must include the selection of bioresources from the waste stream and micro-organisms to prevent waste disposal and prevent the creation of something new to use as a carbon or heteroatom source in the synthesis. Therefore, it is expected that syntheses will use green principles as much as possible in the future. Additionally, syntheses should be efficient in terms of reducing reaction waste, energy, and atomic economy. This type of synthesis must include the selection of bioresources from the waste stream and micro-organisms to prevent waste disposal and prevent the creation of something new to use as a carbon or heteroatom source in the synthesis. The synthesis should use inherently benign and nonhazardous chemistry, which facilitates the prevention of accidents and product toxicity. Bioresource-derived QDs have become the best solution for many applications for a long period of time by ensuring that the synthesis is forward-looking and as green as possible. Since bioresource-derived QDs have been seen to work with microcapacitors/supercapacitors, both in batteries and in targeted solar cells, it stands to reason that bioresource-derived QDs are excellent candidates in the field of energy generation and storage. Typically, bioresource-derived QDs help maintain the cycling stability of electrode materials and shorten charge transfer paths due to their crystal structure and improved surface properties. Considering the advantages and limitations mentioned in the text, it is expected that more research will be conducted to provide high energy density and stable electrodes for energy generation and storage applications in a cost-effective and environmentally friendly design using bioresource-derived QDs. Although QDs play a role in energy generation and storage applications, several critical problems and obstacles have yet to be addressed to fully understand the underlying mechanism, process, and important knowledge of bioresource-functionalized QDs. First, it remains difficult to obtain bioresource-functionalized QDs with high quantum yields. Therefore, future research efforts should focus on chemical and photostability and enhancing high quantum yields. Second, applied research should simultaneously focus on the selectivity, quality improvement, and robustness of QDs with bioresource functionality for energy-oriented platforms. We anticipate that simpler, more revolutionary, and cost-effective green methods, and promising energy applications will be developed in the near future to better exploit the potential of these important quantum materials.

## Figures and Tables

**Table 1:** The properties, advantages, and disadvantages of each method.

Method	Advantages	Disadvantages	Properties	Ref.
microwave	most effective and least time-consuming methods; high efficiency and homogeneous heating	poor control over size, microwave radiation is harmful to human beings	casein dots had high QY of 18.7% and size of 7 nm	[32,33,34,35,36]
hydrothermal	simple to operate, controllable and nontoxic	poor control over size and presence of impurities.	papaya carbon source produced blue colored CDs with a 2−6 nm size and QY of 18.98%; peach extract produced blue nitrogen doped with particle size of 8 nm and a QY of 15%	[37,38,39,40,41,42]
ultrasonic synthesis	process is simple, easy to control, and promotes crystal structures	transformations of the dots, poor size control, and a long reaction time	food waste dots showed a high degree of solubility in water, a narrow band of PL emission (400−470 nm), a size of 4 nm and excellent photostability	[43,44,45,46]
chemical oxidation	very hydrophilic and variable emission, and effective and facile method suitable for large scale; process is easily modified, and surface state is tunable	harsh chemical may be used and biotoxicity of the products is increased	biowaste synthesized dots had an average particle size of 5−6 nm and the QY < 2%	[47,48,49]

**Table 2:** List of micro-organisms (bacteria, yeast, and fungi) through the production of QD and various types of microbial QDs.

	QDs	Optimization of Factors	Microorganisms	Ref.
Fungi	ZnS	-	*Penicillium* sp.	[80]
	CdTe	-	*Fusarium oxysporum*	[81]
	CQD	Concentration	*P. chrysogenum*	[82]
	CdS	Reaction time	*F. oxysporum* f. sp. *lycopersici*	[83]
	PbSe	-	*Aspergillus terreus*	[84]
	MoS_2_	Concentration	*Trichoderma viride*	[85]
	ZnS	Reaction time, temperature, pH	*Aspergillus* sp.	[86]
Bacteria	CdS	CdSO_4_ concentration, temperature, time and pH	*P. chlororaphis* CHR05	[87]
	Carboxylated graphene quantum dots (CGQDs)	Concentrations	*E. coli*	[88]
	CdTe–Rocephin QD complex	-	*E. coli*	[89]
	CQDs	-	*E. coli*	[90]
	CdS	pH	*Acidithiobacillus ferrooxidans*, *A. thiooxidans* and *A. caldus*	[91]
	GQDs-M	-	*Shewanella decolorationis* S12	[92]
	CdSe	-	*Pseudomonas aeruginosa*	[93]
	MoS_2_	-	*E. coli*, *S. aureus*	[94]
	CdTe	-	*E. coli*	[95]
Yeast	CdS	-	*Saccharomyces cerevisiae*	[96]
	CdSe	Different concentrations of Na_2_SeO_3_ and CdCl_2_ and pH	*Rhodotorula mucilaginosa*	[97]
	GQDs	Dose-dependent	*S. cerevisiae* and H9c2 cell line	[98]
	CdSe	Effect of S. cerevisiae growth phase, selenite concentration, cadmium concentration, effects of selenite and cadmium incubating time	*S. cerevisiae*	[99]
	CQDs	-	*Saccharomyces cerevisiae*	[100,101]
	ZnS	Reaction time and different concentrations of yeast biomass and ZnSO_4_	*S. cerevisiae* MTCC 2918	[102]

**Table 3:** Characteristics of QDs and the characterization tools of QDs biosynthesized from bacteria.

QDs	Organism	Characterization Tools	Characteristics of QDs		Ref.
			Size	Shape	
CdS CdS/CdSe	*E. coli*	UV-vis, DLS, HR-STEM, TEM, EDX, FTIR, and fluorescence spectroscopy	12 nm 17 nm	Spherical Spherical	[109]
Graphene	*E. coli*	AFM, HRTEM, UVvis absorption, FT-IR, XPS	3–8 nm	-	[110]
ZnS	*Clostridiaceae* sp.	XRD, EDX, TEM, FTIR, PL and UV	3.34 ± 0.65 nm	Spherical	[111]
Zn/rifampicin/Tf	*Mycobacterium smegmatis*	UV/Vis-spectroscopy, TEM, FTIR, photoluminescence, XRD, XPS and NMR	10 nm	Spherical	[112]
CdTe	*E. coli*	Raman, mass spectrometry, absorption, and fluorescence spectroscopy and fluorescence microscopy	-	-	[113]
Ag/In/S	*Candida albicans*	TEM, XRD, UV-Vis	9.5–10 nm	Spherical	[114]
CdSe	*Providencia vermicola*	UV-vis, FTIR XRD, TEM, and EDX	2–4 nm	Cubic	[115]
ZnO	*E. coli*	X-ray, FTIR, MIC	3–7 nm	Spherical	[116]
CdS	*Pseudomonas fragi*	DSL, AFM, TEM, XRD, XPS, UV-vis and fluorescence emission spectroscopy	2–16 nm	Spherical	[117]

**Table 4:** GQDs applied to MSCs and SCs.

	Electrode Substance	Cycle Stability	Electrolyte	Operating Voltage (V)	Ref.
MSCs	GQD//MnO_2_	-	0.5 M Na_2_SO_4_	1	[161]
	GQD//GQD	97.8%, 5000 cycles	0.5 M Na_2_SO_4_	1	[158]
	GQD//PANI	97.3%, 1500 cycles	0.5 M Na_2_SO_4_	0.9	[162]
SCs	GQD-3DG//GQD-3DG	90%, 5000 cycles	1 M H_2_SO_4_	0.8	[163]
	GEAC//GEAC	100%, 10,000 cycles	Alkaline electrolyte	1	[164]
	CoDC-0.5//CoDC-0.5	90%, 10,000 cycles	6 M KOH	1	[165]

## Data Availability

All data generated or analyzed during this study are included in this published article.
